# Genome‐wide analysis reveals demographic and life‐history patterns associated with habitat modification in landlocked, deep‐spawning sockeye salmon (*Oncorhynchus nerka*)

**DOI:** 10.1002/ece3.8040

**Published:** 2021-08-23

**Authors:** Farida Samad‐zada, Brett T. van Poorten, Shannon Harris, Lyse Godbout, Michael A. Russello

**Affiliations:** ^1^ Department of Biology University of British Columbia Kelowna BC Canada; ^2^ Applied Freshwater Ecology Research Unit British Columbia Ministry of Environment and Climate Change Strategy Vancouver BC Canada; ^3^ School of Resource and Environmental Management Simon Fraser University Burnaby BC Canada; ^4^ Pacific Biological Station, Fisheries and Oceans Canada Nanaimo BC Canada

**Keywords:** ecotype variation, fragmentation, life‐history trait polymorphisms, restoration, salmonids, sex bias

## Abstract

Human‐mediated habitat fragmentation in freshwater ecosystems can negatively impact genetic diversity, demography, and life history of native biota, while disrupting the behavior of species that are dependent on spatial connectivity to complete their life cycles. In the Alouette River system (British Columbia, Canada), dam construction in 1928 impacted passage of anadromous sockeye salmon (*Oncorhynchus nerka*), with the last records of migrants occurring in the 1930s. Since that time, *O. nerka* persisted as a resident population in Alouette Reservoir until experimental water releases beginning in 2005 created conditions for migration; two years later, returning migrants were observed for the first time in ~70 years, raising important basic and applied questions regarding life‐history variation and population structure in this system. Here, we investigated the genetic distinctiveness and population history of Alouette Reservoir *O. nerka* using genome‐wide SNP data (*n* = 7,709 loci) collected for resident and migrant individuals, as well as for neighboring anadromous sockeye salmon and resident kokanee populations within the Fraser River drainage (*n* = 312 individuals). Bayesian clustering and principal components analyses based on neutral loci revealed five distinct clusters, largely associated with geography, and clearly demonstrated that Alouette Reservoir resident and migrant individuals are genetically distinct from other *O. nerka* populations in the Fraser River drainage. At a finer level, there was no clear evidence for differentiation between Alouette Reservoir residents and migrants; although we detected eight high‐confidence outlier loci, they all mapped to sex chromosomes suggesting that differences were likely due to uneven sex ratios rather than life history. Taken together, these data suggest that contemporary Alouette Reservoir *O. nerka* represents a landlocked sockeye salmon population, constituting the first reported instance of deep‐water spawning behavior associated with this life‐history form. This finding punctuates the need for reassessment of conservation status and supports ongoing fisheries management activities in Alouette Reservoir.

## Graphical abstract

Human‐mediated habitat fragmentation in freshwater ecosystems can negatively impact genetic diversity, demography, and life history of native biota, while disrupting the behavior of species that are dependent on spatial connectivity to complete their life cycles. Here, we investigated the genetic distinctiveness and population history of sockeye salmon (*Oncorhynchus nerka*) in Alouette Reservoir in British Columbia, Canada, using genome‐wide SNP data collected for resident and migrant individuals, as well as for neighboring populations within the Fraser River drainage. We found clear evidence that Alouette Reservoir resident and migrant individuals are genetically distinct from other *O. nerka* populations in the Fraser River drainage, but are not differentiated from each other, likely constituting the first reported instance of deep‐spawning behavior associated with this life‐history form and suggesting the need for reassessment of conservation status.

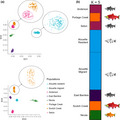

## 1 INTRODUCTION

Life‐history traits, genetic diversity, and structure of wild populations are frequently influenced by anthropogenic stressors, such as human‐induced landscape modifications, habitat loss, and fragmentation (Almeida‐Gomes & Rocha, 2015; Arantes et al., 2019; Boyle et al., 2012; Haag et al., 2010; Roberts et al., 2013). In freshwater ecosystems, water control structures such as dams can restrict spatial habitat connectivity leading to a broad range of consequences, both at the inter‐ and intra‐specific levels (Cooke et al., 2012). For instance, the loss of top predators due to river impediments can inhibit nutrient cycling between different habitats, as well as disrupt the trophic cascade within the lacustrine system (Mattocks et al., 2017). In addition to ecological impacts, connectivity disruption can: (a) lower effective population sizes, increase inbreeding, decrease genetic diversity (Coleman et al., 2018), and cause genetic homogenization (Baggio et al., 2018); (b) skew reproductive success (Maekawa & Koseki, 2001); (c) influence life‐history strategies (Morita et al., 2000); (d) alter population structure (Whiteley et al., 2013); (e) lead to local adaptation (Fraser et al., 2014); and (f) result in extirpation (Morita et al., 2019) and the loss of biodiversity (Liermann et al., 2012). Species that exhibit anadromy are dependent on both freshwater and marine habitats to complete their life cycles and are therefore especially vulnerable to connectivity disruptions (Junge et al., 2014).

The Pacific Northwest is home to many anadromous species, including several salmonids, among which sockeye salmon (*Oncorhynchus nerka*) is particularly notable for its life‐history variation. *O. nerka* is comprised of two major migratory forms: anadromous sockeye salmon (hereafter referred to as “sockeye salmon”) and nonanadromous, resident kokanee (hereafter referred to as “kokanee”), which are further subdivided into ecologically divergent reproductive ecotypes (Quinn, 2005; Taylor et al., 1996). Due to their migratory lifestyle, sockeye salmon provide marine‐derived nutrients to riparian ecosystems that are linked to increases in lake productivity and terrestrial vegetation (Chen et al., 2011; Gende et al., 2002; Quinn et al., 2018; Willson & Halupka, 1995). Pacific salmon are also deeply valued by some First Nations, as for thousands of years *Oncorhynchus* spp. have been a traditional source of sustenance and trade, while serving important cultural and spiritual roles within the communities (Garner & Parfitt, 2006; Jacob et al., 2010). In addition, Pacific salmon constitute exceptionally valuable fisheries, contributing $4.8 billion annually in total economic output in the United States and Canada alone (Gislason et al., 2017). Despite the ecological, cultural, and economic importance of *O. nerka*, the species has experienced significant declines, with many populations currently at risk of extirpation (Gustafson et al., 2007; Rand et al., 2012).

Alouette Reservoir, located in the lower Fraser River drainage in British Columbia, Canada, historically supported populations of all Pacific salmon, including sockeye salmon, but the construction of a dam in 1928 to divert water for hydroelectricity blocked passage to and from the ocean, functionally landlocking *O. nerka* in the newly formed reservoir (Foerster, 1930; Hirst, 1991). The last records of sockeye salmon date back to the 1930s, and the population was first described as kokanee in 1951 (Godbout et al., 2011). Following detection of *O. nerka* juvenile downstream migrants during an intentional experimental water release over the spillway in November 2005, an initiative to restore sockeye salmon in Alouette Reservoir was proposed (Baxter & Bocking, 2006). In 2007 and 2008, *O. nerka* adult upstream migrants were discovered at the base of the Alouette Dam for the first time since initial extirpation (Balcke, 2009). Returning migrants were then transferred above the dam and into the reservoir (Balcke, 2009). Mitochondrial and nuclear microsatellite DNA analyses in combination with otolith microchemistry showed that returning adults were the progenies of resident *O. nerka* from Alouette Reservoir (Godbout et al., 2011). Furthermore, the low diversity at nuclear microsatellites and the fixation of a single mitochondrial DNA haplotype suggested evidence for a recent population bottleneck (Godbout et al., 2011). A subsequent microsatellite‐based study also indicated that Alouette Reservoir *O. nerka* underwent a recent reduction in effective population size in contrast to what was found for populations in neighboring watersheds (Samarasin et al., 2017). Interestingly, both resident and migrant individuals in the Alouette watershed are distinguished morphologically and behaviorally from typical *O. nerka* found elsewhere; during the spawning season, they exhibit a characteristic black or dark olive coloration and build redds at depths of 10–105 m (34 m median depth) below the lake surface (Hébert, 2019). Additionally, resident *O. nerka* have been detected spawning at these depths; although no migrant *O. nerka* were observed in the process of spawning, detection of migrant individuals at the same depth during peak spawning period suggests that migrant Alouette *O. nerka* are likely deep‐spawning as well (Hébert, 2019). This coloration and behavior are in contrast to exceedingly more common shore/beach‐ and stream/river‐spawning *O. nerka* populations that typically exhibit dull to bright pink coloration and spawn less than a meter below the water surface (Quinn, 2005).

In 2016, the Fish and Wildlife Compensation Program identified sockeye salmon restoration in Alouette Reservoir to be of critical importance (Borick‐Cunningham, 2018). However, one persistent challenge is the low proportion of juveniles that undergo smoltification, which is a set of physiological, behavioral, and morphological changes that typically facilitates transition to a saltwater environment (Quinn, 2005). It remains unclear whether Alouette Reservoir is home to two populations (sockeye salmon and kokanee), or whether the life‐history difference represents variation within a single population. This uncertainty persists, in part, due to the lack of records on ecotype variation in Alouette *O. nerka* prior to dam construction. Van Poorten et al. (2018) suggested that pre‐impoundment origin of the Alouette population could be either sockeye salmon that are now landlocked by the dam, or kokanee that previously coexisted with sockeye salmon. Determining whether the contemporary Alouette *O. nerka* population is comprised of one or multiple ecotypes has implications for fisheries management, particularly related to the appropriateness and ultimate success of sockeye salmon restoration efforts.

**FIGURE 1:**
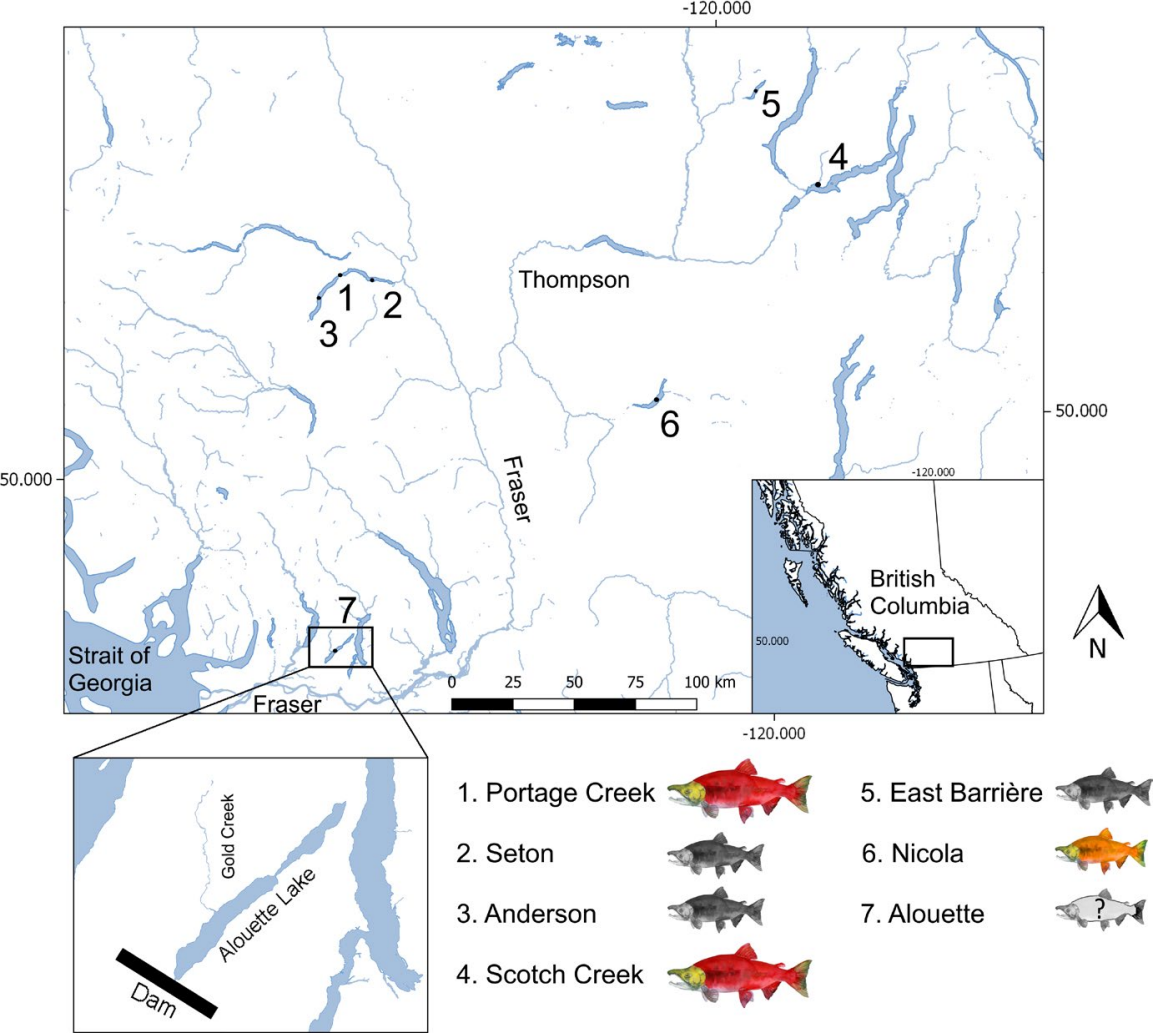
Fraser Basin watershed map, with numbered points indicating lake and creek locations, corresponding to *Oncorhynchus nerka* populations used in this study. Large red fish represent anadromous sockeye, small orange fish represent stream‐spawning kokanee, small black fish represent deep‐spawning kokanee, colorless fish with a question mark represent the ambiguous Alouette population. Fish illustrations are a courtesy of Eileen Klatt. Map produced using QGIS.org (2020), QGIS Geographic Information System, Open Source Geospatial Foundation Project (http://qgis.org). Shapefiles were retrieved from BC Data Catalogue (https://catalogue.data.gov.bc.ca/dataset)

To help fill existing knowledge gaps, we used genotyping‐by‐sequencing of in‐lake, juvenile downstream migrant and adult upstream migrant individuals to investigate the genetic distinctiveness and population history of resident and migrant forms of Alouette *O. nerka* relative to each other and to sockeye salmon and kokanee populations across the Fraser River drainage (Figure 1). In addition, we tested for evidence of adaptive population divergence between resident and migrant individuals in the Alouette system to specifically investigate if there is a genetic basis to migratory behavior. Together, these two objectives afford broader insights regarding how artificial impoundments may shape evolutionary trajectory, life‐history traits, and population structure of recently landlocked *O. nerka*, while providing information for guiding fisheries management.

## 2 METHODS

### 2.1 Study site

Alouette Reservoir (49.3337°N, 122.4181°W), located in British Columbia, Canada, is a small oligotrophic system (area: 16.6 km^2^, maximum depth point: 152 m, dam present) that is comprised of two connected basins, where the southern basin flows into the Alouette River (Plate et al., 2014) (Figure 1). Construction of the Alouette Dam in 1928 in the lower Alouette River isolated the basin, creating the reservoir and preventing salmonid migration. The reservoir has been subject to a nutrient restoration program beginning in 1999, resulting in substantial population growth of *O. nerka* (Harris et al., 2010; Scott et al., 2017; Vainionpaa et al., 2020; van Poorten et al., 2018).

### 2.2 Sample collection

Alouette deep‐spawning resident *O. nerka* (*n* = 68; 43 males/25 females) were sampled in September 2018 from 11 stations located across the reservoir (hereafter referred to as “residents”). Fish were caught with gill nets (stratified to depths of 0, 10, 15, and 20 m) in the pelagic or nearshore habitat. Captured fish were measured [fork length (FL) and mass], sexed, and assessed for life stage via dissection. Operculum punches were taken and stored in vials with 100% ethanol. In addition, Alouette River adult upstream migrant (*n* = 85; no sex data available) and juvenile downstream migrant (*n* = 26; no sex data available) *O. nerka* individuals were provided by Fisheries and Oceans Canada that were originally sampled from 2009 to 2019 (hereafter collectively referred to as “migrants”). These samples consisted of a combination of muscle tissues, operculum tissues, and fins preserved in 100% ethanol.

To allow for broader comparative analyses, we also obtained samples or previously published data from other *O. nerka* populations of known ecotype from locations across the Fraser River drainage, including deep‐spawning kokanee [East Barrière Lake (28.3 km^2^ area, 100 m maximum depth), Seton Lake (24.3 km^2^ area, 151 m maximum depth), Anderson Lake (28.3 km^2^ area, 215 m maximum depth)], stream‐spawning kokanee [Nicola Lake (62.2 km^2^ area, 55 m maximum depth)], and lake‐type sockeye salmon (Scotch Creek, Portage Creek).

East Barrière Lake deep‐spawning kokanee individuals (*n* = 31) were sampled in November 2019. Fish were caught in the pelagic zone using gill nets. Operculum punches were taken and stored in vials with 100% ethanol.

Scotch Creek sockeye salmon individuals (*n* = 25) were sampled as carcasses in September 2019. Muscle tissue was obtained from carcasses and preserved in 100% ethanol.

Nicola Lake kokanee tissue samples (*n* = 25) were collected by trawl at the time of spawning in the Upper Nicola River in September–October 2012. See Acknowledgments for the providers of all tissue samples used in this study.

For Portage Creek sockeye salmon (*n* = 23), and Anderson Lake (*n* = 23) and Seton Lake (*n* = 22) deep‐spawning kokanee samples, we used previously published data from Veale and Russello (2017b). The full sample distribution is summarized in Figure 1, Table 1, and Table A1.

**TABLE 1:** Sample size, ecotype and diversity statistics of the eight *Oncorhynchus nerka* populations

Population	Morphs	Spawner type	*N*	*N_e_* (95% CI)	*H_e_*	*H_o_*	*F_IS_*
Anderson	Kokanee	Deep‐spawning	22	2,120.8 (1,645.0–2,982.0)	0.2646	0.2683	−0.0101
Portage Creek	Sockeye	Stream‐spawning	23	892.0 (800.8–1,006.5)	0.2662	0.2672	−0.0037
Seton	Kokanee	Deep‐spawning	23	1,331.9 (1,142.8–1,595.5)	0.2671	0.2701	−0.0104
Alouette	Resident	Deep‐spawning	61	564.1 (550.9–578.0)	0.2694	0.2731	−0.0060
Alouette	Migrant	Deep‐spawning^a^	102	794.6 (778.4–811.4)	0.2650	0.2460	0.0682
East Barrière	Kokanee	Deep‐spawning	31	909.1 (829.7–1,005.3)	0.2337	0.2448	−0.0357
Scotch Creek	Sockeye	Stream‐spawning	25	1,856.1 (1,530.5–2,356.5)	0.2647	0.2647	0.0015
Nicola	Kokanee	Stream‐spawning	25	2,962.4 (2,011.6–5,608.2)	0.2013	0.2029	−0.0052

Abbreviations: CI, confidence interval; *F_IS_*, inbreeding coefficient; *H_e_*, expected heterozygosity; *H_o_*, observed heterozygosity; *N*, sample size; *N_e_*, effective population size.

^a^ See Section 1 for spawning information of migrant Alouette *O. nerka*.

### 2.3 Library preparation

Genomic DNA was extracted from operculum or muscle tissue using the Qiagen DNeasy Blood and Tissue Kit (Qiagen) following the manufacturer's protocol with the addition of 4 μl of 100 mg/ml 7000 U RNase (Qiagen) prior to ethanol precipitation. We used restriction site‐associated DNA sequencing (RADseq) to simultaneously identify and genotype single nucleotide polymorphisms (SNPs) within the processed *O. nerka* samples. Specifically, we employed a RADseq protocol following Baird et al. (2008) as modified in Lemay and Russello (2015) in order to ensure direct connectivity with a broader dataset generated by Veale and Russello (2017b). Overall, we constructed six libraries that included 260 unique individuals, in addition to 12 within library and seven between library replicates (Table A1). Replicates were added to allow for estimation of genotyping error rates and potential library effects (Tintle et al., 2009). Genomic DNA was digested using the *Sbf1* restriction enzyme and each individual in a library was assigned a unique six nucleotides long barcode. Shearing was performed using a Bioruptor^®^ NGS (Diagenode). Sheared aliquots were cleaned using 1.5X Solid Phase Reversible Immobilization (SPRI) beads and then size‐selected using a Pippin Prep™ (Sage Science) to retain fragments of approximately 500 base pairs. Libraries were PCR‐amplified in parallel by repeating the reaction for 14 cycles. After the final clean up and size‐selection, libraries were sent to the McGill University and Génome Québec Innovation Centre and sequenced using one lane each of Illumina HiSeq 2500 PE125 or Illumina HiSeq 4000 PE150 sequencing (six lanes total).

### 2.4 Genotyping and SNP ascertainment

We combined the newly generated raw sequence reads with those previously collected by Veale and Russello (2017b) for the individuals from Anderson Lake, Seton Lake, and Portage Creek (Table 1). Raw paired‐end reads were demultiplexed and trimmed to 100 bp via the *process radtags* command in STACKS v2.41 (Catchen et al., 2011). Identical reads generated due to PCR amplification were removed using the *clone filter* command in STACKS v2.41 (Catchen et al., 2011). Processed and filtered reads were interleaved and aligned to a reference genome (*Oner_1*, GenBank Assembly Accession ID: GCA_006149115.1; Christensen et al., 2020) using the *bwa mem* algorithm in BWA (Li & Durbin, 2009). The resulting bam files were sorted using SAMtools v1.9 (Li et al., 2009) and used to generate loci and call SNPs via the *gstacks* command in STACKS v2.41 (Catchen et al., 2011). Next, we processed the resulting loci through the *populations* module in STACKS v2.41 (Catchen et al., 2011), calculated mean coverage data per individual using VCFtools v0.1.15 (Danecek et al., 2011), and removed individuals with mean coverage lower than 6x. We then performed a sensitivity analysis on the retained individuals by running the *populations* module in STACKS v2.41 (Catchen et al., 2011) with a varying set of parameters to determine the optimal set for SNP ascertainment. Based on the sensitivity analysis (Table A2), we only retained loci observed in 80% (r80) or more individuals within a population and present in all eight populations, with a minimum minor allele frequency of 0.05 and maximum observed heterozygosity of 0.50. Additionally, *‐‐write‐single‐snp* flag was used to retain only one SNP per locus to decrease the effects of linkage disequilibrium *(‐‐r 0.8, ‐‐p 8, ‐‐min‐maf 0.05, ‐‐max‐obs‐het 0.5, ‐‐write‐single‐snp*). We further processed this dataset through VCFtools v0.1.15 (Danecek et al., 2011) to only include sites with a minimum mean depth of 10x and a maximum mean depth of 100x and exclude sites with more than 10% missing data (‐‐*max‐missing 0.9*). Putative paralogs were identified using the *HDplot* function available from https://github.com/gjmckinney/HDplot (McKinney et al., 2017) and removed using VCFtools v0.1.15 (Danecek et al., 2011). Lastly, we estimated genotyping error rates using a custom python script (https://github.com/bsjodin/genoerrorcalc) and removed replicate individuals from the dataset.

### 2.5 Outlier detection

Given the high false‐positive rates associated with outlier detection approaches and the hierarchical population structure of our dataset, we employed three different analyses including the *F_ST_*‐based approaches implemented in Arlequin v3.5 (Excoffier & Lischer, 2010) and Bayescan v2.0 (Foll & Gaggiotti, 2008), and the principal component analysis (PCA)‐based approach implemented in *pcadapt* (Luu et al., 2017). For Arlequin, we used the hierarchical island model (Slatkin & Voelm, 1991) that allows a higher migration rate between populations within a group than between groups. We performed 20,000 simulations, with the number of simulated demes set to 100, number of simulated groups set to 10, and simulated derived allele frequency set to 0.05. We considered all loci with *p*‐values < 0.01 in the first and last quantile as candidate loci under divergent or balancing selection, respectively. For BayeScan v2.0 (Foll & Gaggiotti, 2008), we used a pairwise approach comparing allele frequencies between all possible pairs of populations, resulting in 28 pairwise comparisons. Additionally, we used this method to detect outliers between combined ecotype datasets using the following comparisons (a) all sockeye salmon populations (Scotch Creek, Portage Creek) versus all kokanee populations (Anderson, Seton, Nicola, East Barrière) and (b) all deep‐spawning kokanee (Anderson, Seton, East Barrière) versus stream‐spawning kokanee (Nicola). Alouette resident and migrant individuals were excluded from the ecotype comparisons given the uncertainty regarding the true ecotype(s) of this population. The analyses were run for 100,000 iterations with 50,000 burn‐in period with Prior Odds set to 10, and loci with *q*‐values less than 0.05 were marked as outliers and removed from the dataset. Lastly, we inferred genetic clusters through analyses of principal components (PCs) using *pcadapt* v4.1.0 (Luu et al., 2017) and Cuttel's rule to infer the most likely number of PCs that explained the genetic structure within the dataset. The resulting *p*‐values were corrected for multiple comparisons using the method of Benjamini‐Yekutieli (2001), and loci with adjusted *p*‐values of less than 0.05 were deemed outliers. We functionally annotated loci that were found in the comparisons of sockeye salmon versus kokanee, as well as deep‐spawning versus stream‐spawning kokanee. Specifically, we used the *blastn* function in BLAST v2.9.0 (Altschul et al., 1990) to compare the locus sequences including outlier SNPs to the *nr* database within the *Oncorhynchus* taxon, accepting hits with an e‐value lower than 1e^−28^ and retaining hits with the lowest e‐value.

Additionally, we specifically examined the genotypes for all Alouette individuals at the sockeye‐kokanee outliers. To test whether genotype counts were significantly different between residents and migrants at each of these outliers, we used Fisher's exact test as implemented in R package stats v3.5.2 (R Core Team, 2018).

### 2.6 Alouette migrant–resident outlier detection

We also conducted outlier detection directly for the Alouette migrant versus resident individuals using BayeScan v2.0 (Foll & Gaggiotti, 2008) and *pcadapt* v4.1.0 (Luu et al., 2017), and the same parameters as for the full dataset (see Section 2.5). In addition, we conducted a GWAS analysis to investigate the relationship between SNPs and phenotype (i.e., resident or migrant) using the mixed linear model implemented in the R program GAPIT v3.0 (Lipka et al., 2012; Wang & Zhang, 2020). This analysis was conducted on a reduced dataset of 6,775 SNPs that successfully mapped to linkage groups (*Oner_1*, GenBank Assembly Accession ID: GCA_006149115.1, Christensen et al., 2020), rather than to unplaced scaffolds (“UN”). Relatedness between pairs of individuals was accounted for by calculating a kinship matrix (VanRaden, 2008); however, the number of PCs was set to 0 given the absence of population structure in Alouette *O. nerka* based on STRUCTURE and PCA (see Section 3). The FDR‐corrected threshold was set to 0.05, and all SNPs below that threshold were considered significant.

### 2.7 Population genetic analyses

To construct a putatively neutral dataset, we removed any locus identified as an outlier in any comparison. Following outlier removal, we removed loci that significantly (‐h 0.05) deviated from Hardy–Weinberg equilibrium in 50% or more of the populations using the filter_hwe_by_pop.pl script available from https://github.com/jpuritz/dDocent/tree/master/scripts. Using the resulting putatively neutral SNP dataset, we calculated inbreeding coefficients (*F_IS_*), observed (*H_o_*), and expected (*H_e_*) heterozygosity per locus following Nei (1987) and averaged across loci for each population using the *basic.stats* command within the R package *hierfstat* v0.04‐22 (Goudet & Jombart, 2015). We also estimated effective population sizes (*N_e_*) for each population using the linkage disequilibrium method (Waples & Do, 2008) as implemented in NeEstimator v.2 (Do et al., 2014), with the minimum allele frequency set to 0.05. To calculate levels of population differentiation, we calculated Weir and Cockerham’s (1984) *θ* between all pairs of populations using 1,000 permutations in Genetix v.4.05.2 (Belkhir et al., 2004). We visualized obtained pairwise *θ* values via NeighbourNetwork (Bryant & Moulton, 2004) reconstruction using SPLITSTREE v4.0 (Huson & Bryant, 2006).

To evaluate the number of genetic clusters present in our dataset, we used the Bayesian method of Pritchard et al. (2000) as implemented in STRUCTURE v3.4 (Pritchard et al., 2000). Run length was set to 100,000 Markov chain Monte Carlo replicates after a burn‐in period of 100,000 using correlated allele frequencies under an admixture model using the LOCPRIOR option. We varied the number of clusters (*K*) from one to ten, with ten iterations of each. The resulting output was then summarized using STRUCTURE HARVESTER (Earl & vonHoldt, 2012). To infer the optimal *K* value, we employed a combination of the Δ*K* method (Evanno et al., 2005) and the plotting of the log probability of the data (Pritchard et al., 2000) to assess where ln Pr(*X|K*) plateaued (see STRUCTURE manual) and then used CLUMPAK (Kopelman et al., 2015) to visualize the results. Additionally, we conducted a PCA to visualize the relationships among populations using *SNPRelate* v1.14.0 (Zheng et al., 2012).

## 3 RESULTS

### 3.1 Dataset quality

After demultiplexing, trimming and quality filtering, we retained a mean of 7,201,813 reads per sample. Across samples, an average of 94.22% of reads was successfully mapped to the reference genome. After full filtering, 7,709 SNPs were retained for 312 individuals (17 individuals were removed due to insufficient coverage <6x), with a mean depth of 25.27x and mean missing percentage of 3.84%. Mean within‐ and among‐library genotyping error rates were 5.46% and 3.99%, respectively.

### 3.2 Outlier loci detection, mapping, and annotation

For the Fraser River drainage‐wide analyses, Arlequin identified 154 high *F_ST_* outliers and 118 low *F_ST_* outliers. BayeScan detected 253 outliers with a *q*‐value lower than 0.05 across all pairwise population comparisons. The *pcadapt* analysis identified 473 loci with a *p*‐value lower than 0.05 after the Benjamini‐Yekutieli (2001) correction. The first and second principal components showed six distinct clusters, largely associated with geography, where PC1 separated the Alouette individuals from the rest of the populations, and PC2 divided the remaining populations into four clusters (Figure A2). Of the identified outliers, 54 loci were detected in common by all three methods. Additionally, outlier detection between sockeye salmon‐kokanee ecotypes resulted in 14 outliers (Figure A3), four of which were in common with the 54 loci detected by all three methods at the population level. Outlier detection between deep‐spawning and stream‐spawning kokanee resulted in four outliers, with no overlap with the 54 outliers identified in the population‐level analyses or the 14 sockeye‐kokanee outliers. Mapping to the *O. nerka* genome assembly showed that all outlier loci, including the 14 loci divergent between sockeye salmon and kokanee ecotypes, were distributed across different linkage groups. Of the 14 sockeye salmon‐kokanee outlier loci, 12 produced significant annotations (Table 2), six of which overlapped with those found in previous studies (Nichols et al., 2016; Veale & Russello, 2017b). Examining the genotypes of the Alouette Reservoir migrant and resident individuals at the 14 sockeye salmon‐kokanee outlier loci revealed no significant difference (*p*‐value > 0.05) in genotype frequencies between the two groups at 13 of these 14 loci. Genotype frequencies were significantly different (*p*‐value = 0.006815) between residents and migrants only at locus SZNR01029834.1_1048492 (no BLAST annotation available).

**TABLE 2:** IDs and annotations of outliers detected by BayeScan between sockeye salmon and kokanee populations in this study, and corresponding loci ID of sockeye salmon‐kokanee outliers detected in Nichols et al. (2016) and Veale and Russello (2017b)

SNP	Annotation	Nichols et al. (2016)	Veale and Russello (2017b)
SZNR01010580.1_848156^a^	*Oncorhynchus nerka* isolate LRRC9_Ok_shore leucine‐rich repeat‐containing protein 9‐like protein gene, complete cds	RADtag_57884	68810_51
SZNR01019686.1_513507	PREDICTED: *Oncorhynchus kisutch* TIR domain containing adaptor protein (tirap), mRNA		
SZNR01002048.1_136609	PREDICTED: *Oncorhynchus kisutch* proteoglycan 4 (LOC109908567), mRNA	RADtag_66595	
SZNR01024871.1_93859	PREDICTED: *Oncorhynchus nerka* heat shock protein HSP 90‐alpha 1 (LOC115118567), transcript variant X1, mRNA		40949_10
SZNR01027302.1_1918892	PREDICTED: *Oncorhynchus kisutch* proteoglycan 4 (LOC109908567), mRNA		
SZNR01027302.1_1918732	PREDICTED: *Oncorhynchus kisutch* inactive phospholipid phosphatase 7 (LOC109868517), mRNA	RADtag_18513	112822_83
SZNR01029823.1_2579190	PREDICTED: *Oncorhynchus mykiss* uncharacterized LOC110523490 (LOC110523490), ncRNA		
SZNR01029834.1_1048492	PREDICTED: *Oncorhynchus mykiss* uncharacterized LOC110496934 (LOC110496934), ncRNA		
SZNR01004580.1_453594	PREDICTED: *Oncorhynchus kisutch* proline‐rich transmembrane protein 1‐like (LOC109908550), mRNA	RADtag_7544	14428_85
SZNR01004638.1_505442	*Oncorhynchus tshawytscha* follicle‐stimulating hormone beta subunit (FSHbeta) gene, promoter and complete cds		
SZNR01007172.1_549647^a^	PREDICTED*: Oncorhynchus kisutch* stearoyl‐CoA desaturase 5 (LOC109868414), mRNA		
SZNR01007191.1_179523	PREDICTED: *Oncorhynchus nerka* partitioning defective 6 homolog alpha‐like (LOC115134284), mRNA		3833_28
SZNR01010580.1_883229^a^	NA		
SZNR01022265.1_96641	NA		

Note

Populations used in this comparison:

Sockeye salmon: Scotch Creek and Portage Creek.

Kokanee: Nicola, Anderson, Seton and East Barrière Lakes.

^a^ Outliers detected both in the ecotype outlier scan, and multiple pairwise comparison scans between sockeye and kokanee populations.

### 3.3 Alouette migrant–resident outlier detection

**FIGURE 2:**
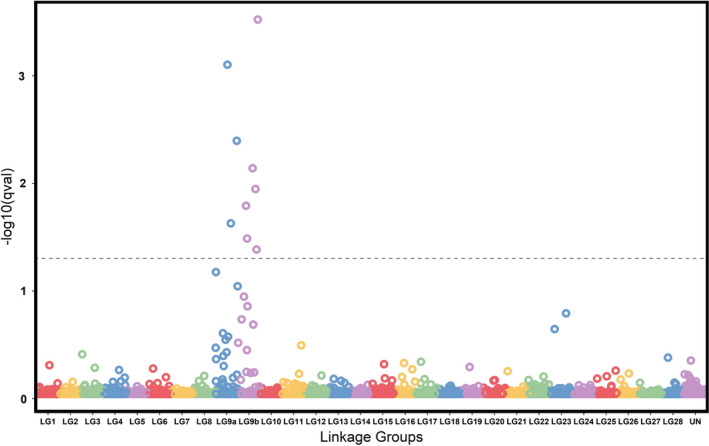
Manhattan plot, representing the −log10(qval) of 7,709 SNPs across 29 linkage groups (LG) and unplaced scaffolds (UN), as calculated by the BayeScan (Foll & Gaggiotti, 2008) outlier scan between resident and migrant Alouette *Oncorhynchus nerka*. The dot‐line corresponds to the *q*‐value of 0.05. Figure produced using *tidyverse* packages *ggplot2*, *dplyr* and *tidyr* (Wickham et al., 2019)

**FIGURE 3:**
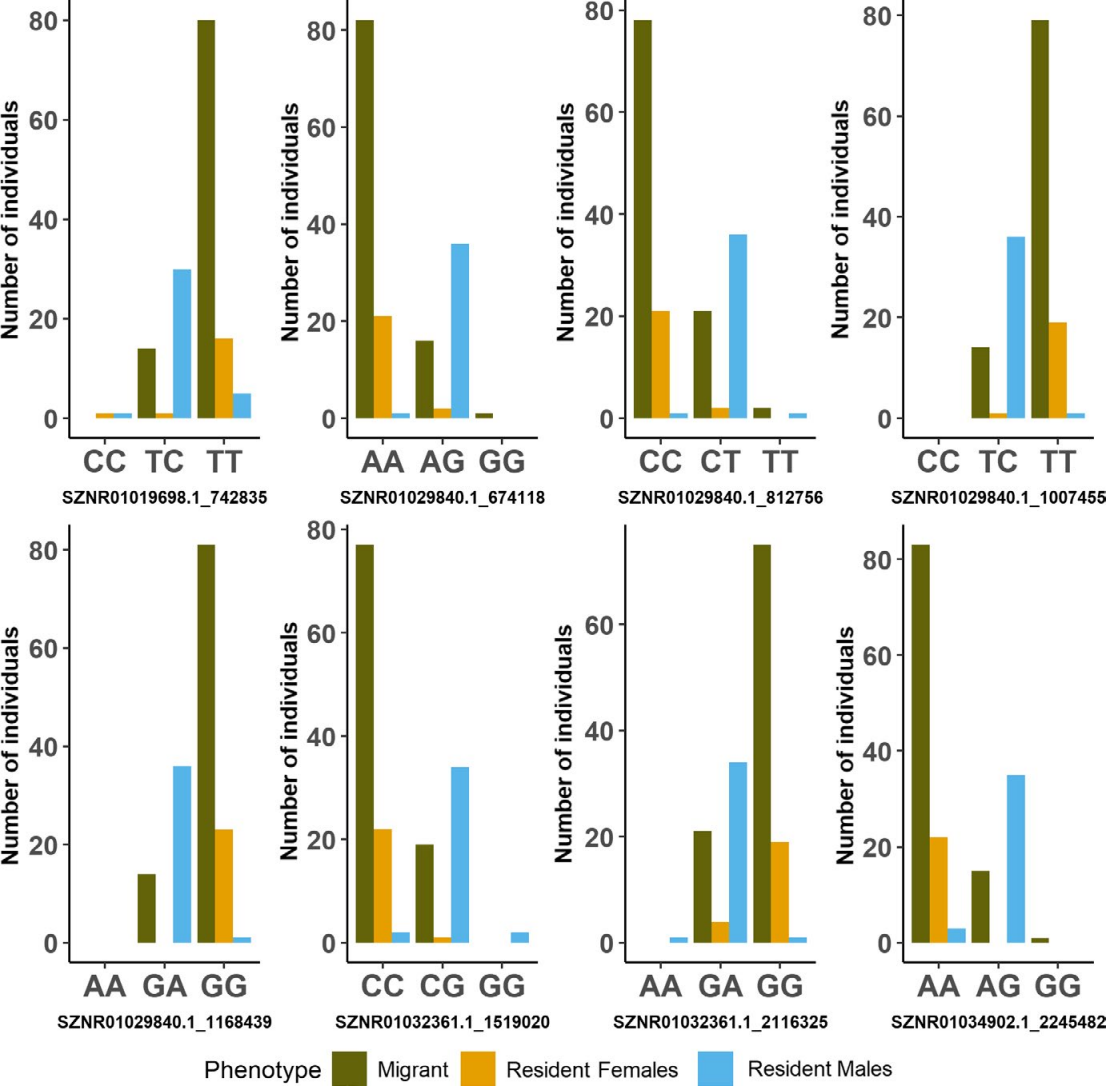
Allele frequencies of the eight high‐confidence outliers detected in comparisons of Alouette Reservoir migrants and residents, compared across three groups: resident female *Oncorhynchus nerka* (*n* = 23), resident male *O. nerka* (*n* = 38), migrant *O. nerka* (*n* = 102). Samples that did not genotype at a particular SNP are not included in this figure. Figure produced using *tidyverse* packages *ggplot2*, *dplyr* and *tidyr* (Wickham et al., 2019)

**FIGURE A1:**
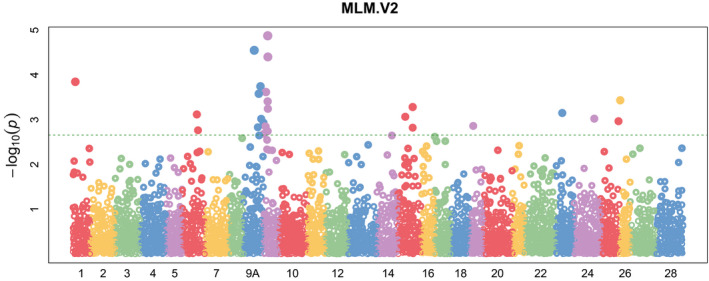
Manhattan plot generated using GWAS Mixed Linear Model (MLM) analysis, using GAPIT v3.0 (Lipka et al., 2012). Numbers on x‐axis correspond to Linkage Groups. V2 corresponds to phenotype (migratory or resident)

**FIGURE A2:**
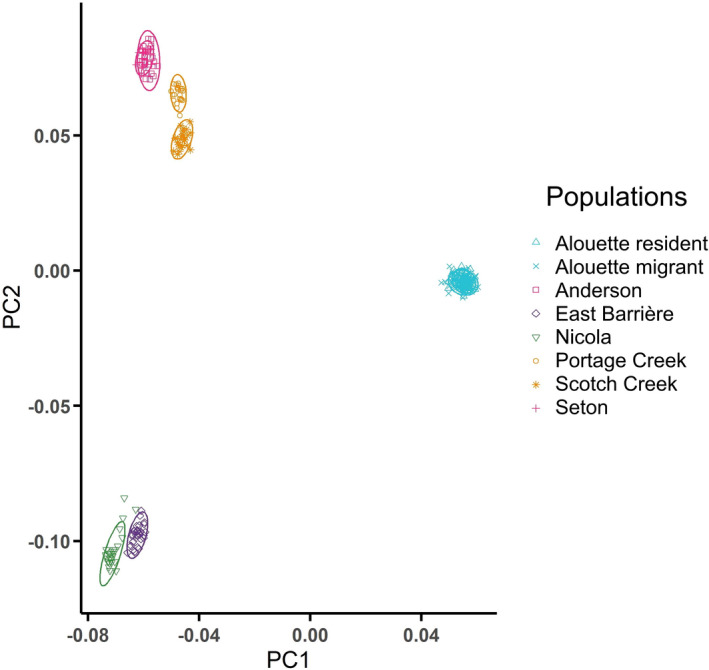
PCA plot used in identification of outliers by *pcadapt*. PC1 explains 16.8% of variance, while PC2 explains 5.9%

**FIGURE A3:**
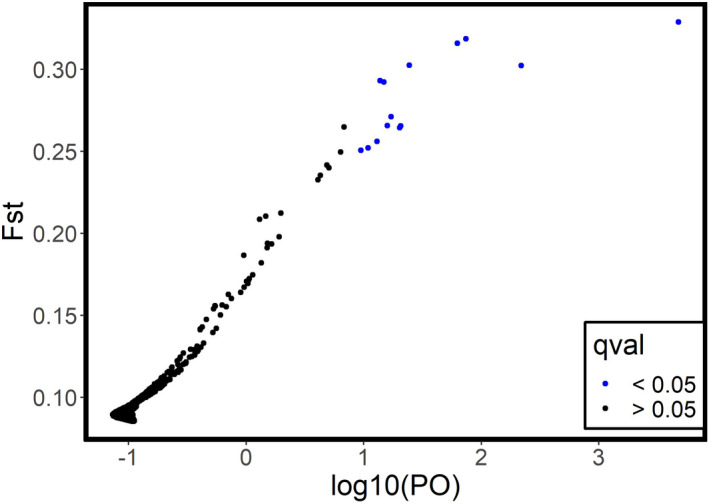
BayeScan plot demonstrating outliers between sockeye and kokanee populations. SNPs highlighted in blue (*n* = 14) correspond to the 14 outliers shown in Table 2

Pairwise BayeScan analysis between Alouette Reservoir migratory and resident individuals detected nine outliers, whereas *pcadapt* analysis identified 48 outliers associated with PC2; eight outliers were found in common between the two analyses. All detected outliers mapped to linkage groups 9a and 9b (Figure 2), which correspond to sockeye salmon sex chromosomes (Christensen et al., 2020). GWAS did not detect any outliers after the FDR correction, however, the Manhattan plot generated by this analysis showed that SNPs distributed across linkage groups 9a and 9b had higher log values (Figure A1). Of the eight outliers detected by both methods, four produced significant annotations (Table A3), but none overlapped with the 14 outliers detected in the basin‐wide sockeye salmon‐kokanee outlier analysis. Genotype frequencies at the eight common outliers were uneven across the Alouette Reservoir individuals, with resident females and migrant individuals exhibiting similar frequencies to each other relative to resident males (Figure 3). Likewise, resident females and migrant individuals exhibited clear heterozygote deficits (*H_o_* = 0.00–0.22; migratory mean *H_o_* = 0.17, resident female mean *H_o_* = 0.06), unlike the levels detected in resident males (*H_o_* = 0.83–0.97; mean *H_o_* = 0.93).

### 3.4 Population genetics

We removed all SNPs that were identified as outliers (*n* = 696) by any of the three above‐mentioned analyses. One additional locus was found to deviate from HWE in more than 50% of the populations and was also removed. Based on this putatively neutral dataset of 7,012 SNPs, *H_o_*, *H_e_*, and *F_IS_* values were similar across the eight populations, with both *H_o_* and *H_e_*, ranging from 0.20 to 0.27 (Table 1). None of the *F_IS_* values significantly differed from 0 across populations, except that of Alouette migrants (Table 1). Nicola Lake kokanee had the highest *N_e_* [2,962.4 (2,011.6–5,608.2)], while both migrant and resident Alouette groups had the lowest *N_e_* [794.6 (778.4–811.4), 564.1(550.9–578.0), respectively] (Table 1).

**FIGURE 4:**
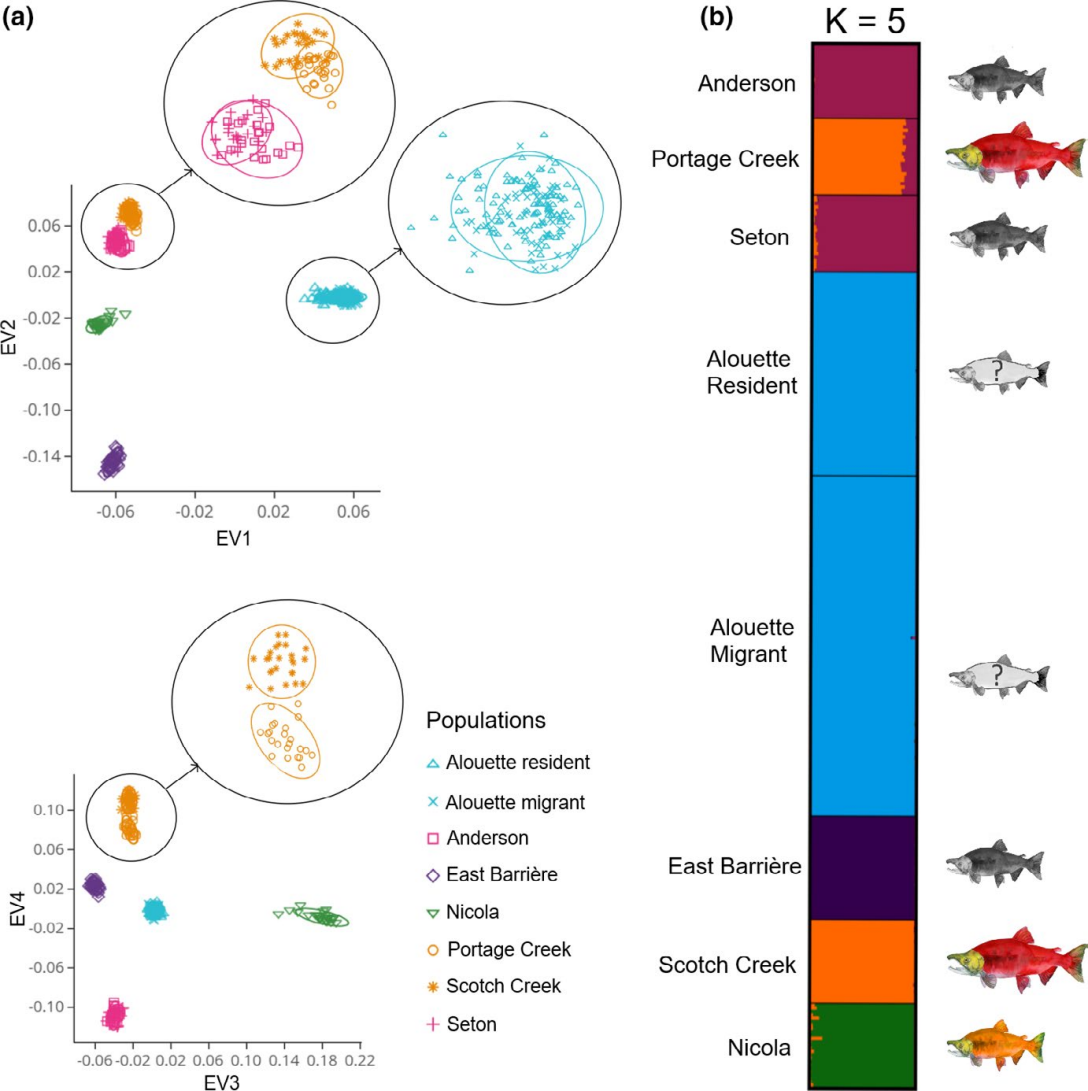
(a) Principal component analysis (PCA) for 312 individuals, produced using 7,012 putatively neutral SNPs. This analysis was conducted in *SNPRelate* v1.14.0 (Zheng et al., 2012). EV1, EV2, EV3, and EV4 explain 37.31%, 12.29%, 11.97%, and 7.76% of the variation, respectively. (b) Results of Bayesian clustering method, as implemented in STRUCTURE v3.4 (Pritchard et al., 2000). Output results represent the optimal *K* value (*K* = 5), as determined by the Δ*K* method (Evanno et al., 2005), as implemented in STRUCTURE HARVESTER (Earl & vonHoldt, 2012). Visualized using CLUMPAK (Kopelman et al., 2015)

**FIGURE A4:**
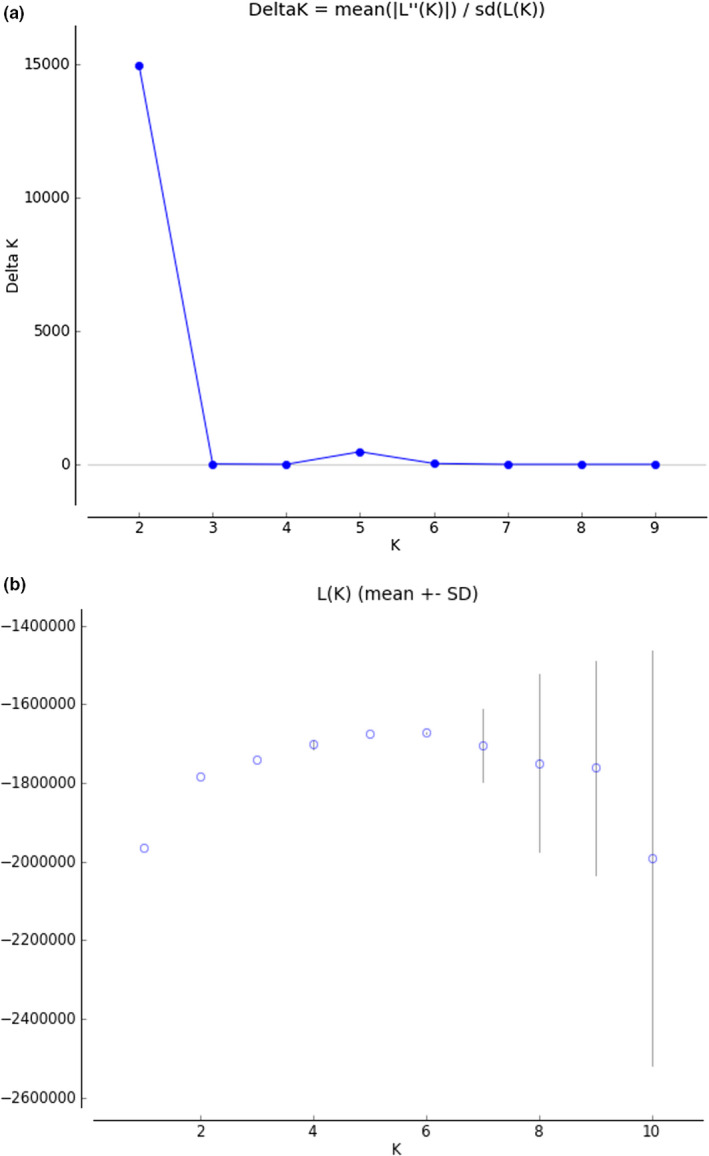
(a) Delta *K* values for the 10 iterations of STRUCTURE with the number of clusters varying from 1 to 10 and (b) ln Pr(*X|K*) probability plot generated using STRUCTURE Harvester

The STRUCTURE analysis revealed evidence for five clusters that best explained the genetic variation within our dataset, largely conforming to geography (Figure 4b, Figure A4 and Table A4). Alouette Reservoir *O. nerka* was identified as a distinct cluster starting from *K* = 2, with both resident and migrant individuals belonging to the same cluster, even with increasing values of *K*. East Barrière kokanee separated from the remaining populations at *K* = 3, and Nicola Lake separated at *K* = 4. At *K* = 5, Anderson Lake and Seton Lake deep‐spawning kokanee formed a cluster, while Portage Creek and Scotch Creek sockeye salmon formed a separate cluster. The Portage Creek and Scotch Creek sockeye salmon did not separate into distinct clusters at any higher values of *K*. None of the populations demonstrated any evidence for further substructure and no admixture was detected between the populations.

The PCA on the neutral dataset also demonstrated evidence for five clusters, with Alouette migrant and resident individuals belonging to the same cluster, regardless of which eigenvectors were plotted (Figure 4a). Eigenvector 1 explained 37% of the variation and separated Alouette migrant and resident individuals from the rest of the populations; eigenvector 2 explained 12.2% of the variation and separated East Barrière Lake. Similar to the STRUCTURE analysis, Anderson Lake and Seton Lake deep‐spawning kokanee populations clustered together regardless of the eigenvectors used. Portage Creek and Scotch Creek sockeye salmon clustered close together when eigenvectors 1 and 2 were plotted; however, they formed two distinct clusters when this was extended to eigenvectors 3 and 4 (Figure 4a).

**FIGURE 5:**
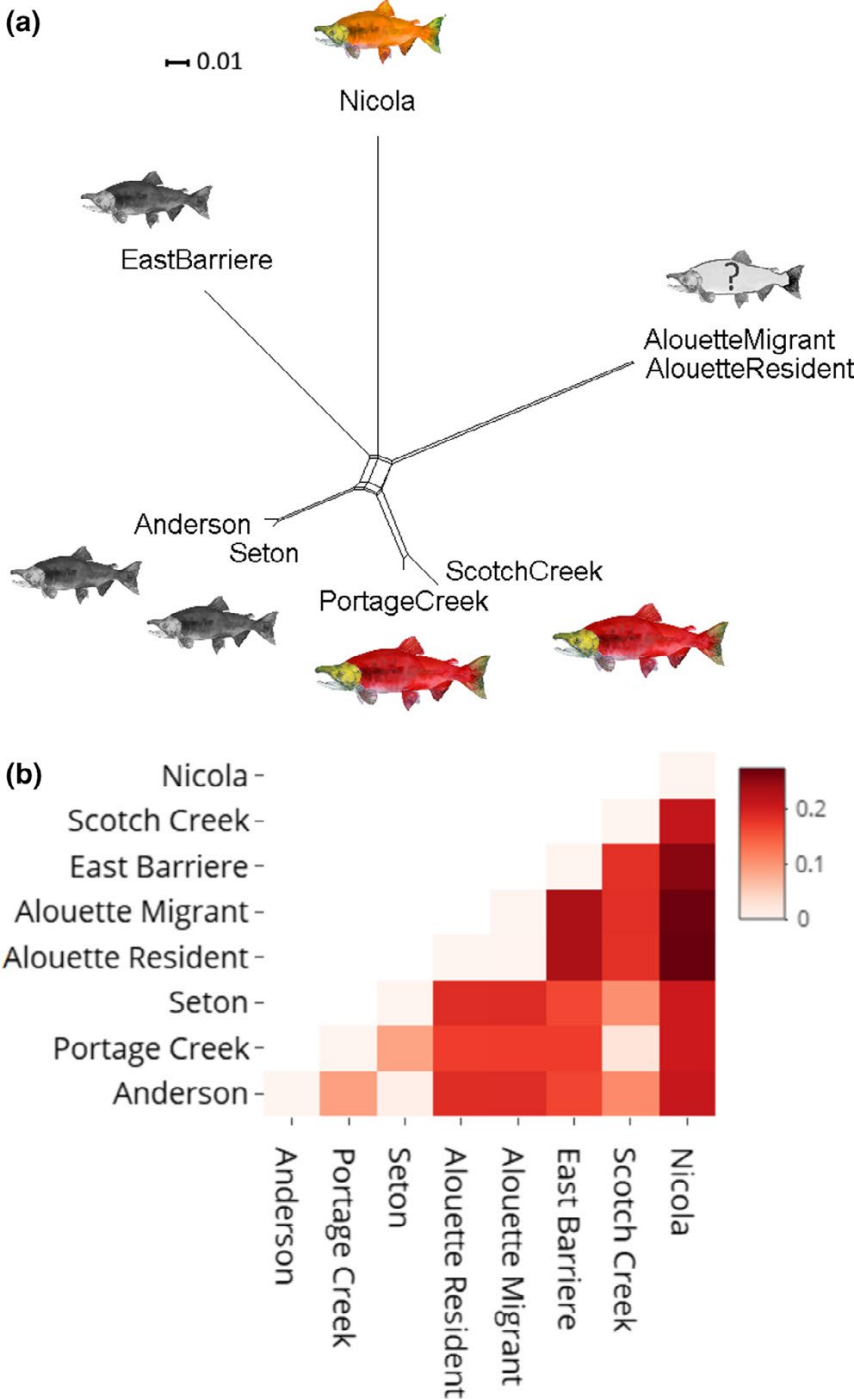
(a) NeighborNetwork (Bryant & Moulton, 2004), based on the Weir and Cockerham’s (1984) pairwise *θ* values calculated in Genetix (Belkhir et al., 2004), using 7,012 putatively neutral SNPs, visualized using SPLITSTREE v4.0 (Huson & Bryant, 2006). (b) Heat map of the *θ* matrix produced using R package *plotly* v4.9.0 (Sievert, 2020). The color scale bar represents pairwise *θ* values

The phylogenetic network based on the neutral dataset did not show any clear separation by ecotype, but provided further evidence for geographic differentiation (Figure 5a). As in the STRUCTURE and PCA, Alouette Reservoir migrant and resident individuals clustered together, as did Portage Creek and Scotch Creek sockeye salmon (Figure 5a). Kokanee populations that were not geographically close to a sampled sockeye salmon population (Nicola Lake stream‐spawning and East Barrière Lake deep‐spawning kokanee) were more isolated, as indicated by longer branch lengths (Figure 5a) and higher pairwise *θ* values (Figure 5b).

## 4 DISCUSSION

Our genome‐wide analyses provide clear evidence that Alouette Reservoir resident and migrant individuals are genetically distinct from other *O. nerka* populations in the Fraser River drainage included in this study, but are not differentiated from each other, likely constituting a single population. Results based on neutral and outlier genotypic variation further suggest that contemporary Alouette Reservoir *O. nerka* represents a landlocked sockeye salmon population, which, to our knowledge, would constitute the first reported instance of deep‐spawning behavior associated with this life‐history form. Taken together, these results offer further insights into sockeye salmon demographic and life‐history variation, while providing important information for guiding ongoing fisheries management in the Alouette watershed.

### 4.1 Geographic differentiation

Our results demonstrated that population structure of sockeye salmon and kokanee across the Fraser River Basin was largely associated with geography, rather than ecotype, consistent with previous findings in this system (Beacham & Withler, 2017; Veale & Russello, 2017b). For example, East Barrière Lake deep‐spawning kokanee and Nicola Lake stream‐spawning kokanee each formed distinct clusters, likely due to the geographic and temporal isolation of these resident populations (Wood et al., 2008). Our results align with previous findings that demonstrated that within the drainage, kokanee inhabiting different lakes are genetically isolated from each other and more closely related to sympatric sockeye salmon populations if present (Beacham & Withler, 2017). Here, the only group within which sockeye salmon and kokanee populations were in direct geographic proximity was Anderson Lake and Seton Lake that are connected by Portage Creek. In this system, deep‐spawning kokanee from Anderson Lake and Seton Lake grouped together and were most closely related to Portage Creek sockeye salmon, consistent with previous studies (Moreira & Taylor, 2015; Veale & Russello, 2017b). Moreover, Portage Creek and Scotch Creek sockeye salmon displayed high genetic affinity despite being located more than 300 km away from each other, exhibiting no evidence of pairwise differentiation (Figure 5b), while forming a single STRUCTURE cluster (Figure 4b) and largely overlapping PCA clusters (Figure 4a). The lack of differentiation at the neutral data may be explained by Portage Creek hatchery supplementation from the Lower Adams River (neighboring Scotch Creek) that aimed to restore the declining sockeye salmon population in the 1950s (Withler et al., 2000). Overall, these patterns were consistent with previous findings by Wood et al. (2008), which showed that within drainages, among population differentiation was lower for lake‐type sockeye salmon than for kokanee.

Alouette Reservoir *O. nerka*, in particular, showed clear separation from the rest of the populations in this study, exhibiting the highest and lowest levels of differentiation from Nicola Lake kokanee and Portage Creek sockeye salmon, respectively (Figure 5). Yet, while Portage Creek and Scotch Creek sockeye salmon showed little to no evidence of genome‐wide differentiation (Figures 2 and 5), Alouette Reservoir *O. nerka* was still significantly differentiated from each (Figure 5b). These reconstructed patterns are not necessarily indicative of the *O. nerka* ecotype in Alouette Reservoir in the absence of the dam, as the substantial pairwise genetic differentiation relative to all other populations in the study could be due to extreme drift (Perrier et al., 2013) given the genetic bottleneck that has been previously reported in this system (Godbout et al., 2011; Samarasin et al., 2017). The role of drift may be further evidenced by the lower *N_e_* in Alouette Reservoir compared to all other populations in this study (Table 1) and also consistent with the low census adult population size (*N_c_* = ~20,000) reported prior to the start of the fertilization program in 1999 that has subsequently increased to ~200,000 individuals in 2019 (Harris et al., 2010).

At a finer level, within‐Alouette Reservoir analyses of migratory and resident individuals revealed no evidence for differentiation based on neutral genome‐wide data (Figures 2 and 5), consistent with a single population. These results mirrored those from a previous microsatellite study which revealed that Alouette Reservoir individuals formed a single genetic cluster, regardless of their migratory tendencies (Godbout et al., 2011). Understanding whether migratory behavior of Alouette individuals has an underlying genetic basis, however, cannot be deciphered using neutral data alone.

### 4.2 Outlier loci and ecotype identification

Identifying genetic mechanisms responsible for parallelisms can help our understanding of the repeatability of evolution, as well as molecular processes that shape phenotypic variation, local adaptation, and life‐history traits (Lee et al., 2014). Outlier locus detection is frequently used for investigating molecular drivers behind parallel phenotypic divergence (Deagle et al., 2012; Perrier et al., 2013; Westram et al., 2014). Moreover, outlier loci can be useful for differentiating between populations that have only recently been isolated and for which neutral variation has not yet coalesced (Russello et al., 2012). Comparison of allele frequencies between all sockeye salmon and kokanee populations within our dataset revealed 14 loci that were significantly differentiated between the two ecotypes. Functional annotations were available for 12 of these SNPs (Table 2); however, we limit our discussion to six robust loci that were also identified as candidates under divergent selection in previous population genomic studies of sockeye salmon and kokanee (Nichols et al., 2016; Veale & Russello, 2017b), or have been detected in multiple comparisons in this study.

SZNR01010580.1_848156 mapped to the leucine‐rich repeat‐containing protein 9 (LRRC9) gene, at which specific genotypes have been previously found to be associated with spawning location (GG = shore‐/beach‐spawning; TT/GT = stream‐/river‐spawning) in both migrant and resident *O. nerka* across the entire distribution (Veale & Russello, 2017a, 2017b). Genotypes at this locus were entirely consistent with the previously known reproductive ecotypes including shore/deep‐spawning kokanee (East Barrière Lake: GG genotype in 31/31 samples; Anderson Lake: GG genotype in 21/23 samples; Seton Lake: GG genotype in 22/22 samples), stream‐spawning kokanee (Nicola Lake: GG genotype in 0/25 samples); and stream‐spawning sockeye salmon (Portage Creek: GG genotype in 0/23 samples; Scotch Creek: GG genotype in 0/25 samples). In Atlantic salmon, LRRC9 is located 142 kb away from the *six6* gene that exhibits signatures of divergent selection with respect to spawning ecotypes and has been associated with age at maturity (Barson et al., 2015) and marine diet specialization (Aykanat et al., 2020). Recent work has further demonstrated significant associations between *six6* and age at maturity in both sockeye salmon and steelhead trout (Waters et al., 2021; Willis et al., 2020).

SZNR01024871.1_93859 is located in the *O. nerka* heat shock protein HSP 90‐alpha gene and was previously identified as an outlier in sockeye salmon‐kokanee comparisons in the Okanagan and Anderson‐Seton‐Portage systems (Veale & Russello, 2017b). Heat shock proteins are molecular chaperones that assist protein folding and stabilization to help cells combat thermal stress; HSP90, in particular, is a highly interactive protein, involved in numerous molecular pathways (Saibil, 2013). Transcriptomic studies found that expression of HSP90 was increased in the gills of chinook salmon, *Oncorhynchus tshawytscha*, in response to increased water temperatures (Tomalty et al., 2015). More broadly, the debilitating effect that increasing water temperature can have on migratory salmon (Crossin et al., 2008) has been associated with changing expression of HSP90 at various periods of migration (Miller et al., 2009).

Two other outliers identified between sockeye salmon and kokanee populations were annotated to regions likely associated with diet. First, SZNR01007172.1_549647 is located in coho salmon, *Oncorhynchus kisutch*, stearoyl‐CoA desaturase 5 gene (SCD‐5). However, this gene annotation was predicted computationally and taken together with studies that show that SCD‐5 has been lost in teleost fishes, it is possible that this SNP is located in another SCD gene (Castro et al., 2011). The exact function of SCD genes in fish is unknown, but previous research found an influence of dietary intake on the expression of some SCD genes, which might differ based on the availability of dietary fatty acids in the fish rearing habitat (Castro et al., 2011). Likewise, SZNR01007191.1_179523 annotated to *O. nerka* partitioning defective 6 homolog alpha‐like mRNA (*par‐6*); *par‐6* homolog expression has been demonstrated to change in Atlantic salmon liver following a dietary switch (Leaver et al., 2008).

The last two sockeye‐kokanee outliers were found in genes that have been related to transition from the marine environment to freshwater, and tissue regeneration. SZNR01004638.1_505442 is located in *O. tshawytscha* follicle‐stimulating hormone beta subunit (FSHbeta) gene. The FSH hormone belongs to the Glycoprotein Hormone Family (GPH), and expression of FSHbeta changes upon transition to freshwater in adult chum salmon (*Oncorhynchus keta*; Kim et al., 2013). In addition, SZNR01002048.1_136609, which mapped to *Oncorhynchus kisutch* proteoglycan 4, has been associated with wound healing (Hirose et al., 2018).

We specifically examined the genotypes of Alouette Reservoir migratory and resident *O. nerka* individuals at the 14 sockeye salmon‐kokanee outliers to further investigate specific associations with different migratory and reproductive behaviors. Overall, migratory and resident individuals demonstrated no significant difference in genotype frequencies across 13 of these 14 loci, further consistent with the hypothesis that Alouette Reservoir *O. nerka* comprise a single population. Of particular note, all resident and migrant individuals were fixed for the GG genotype at SZNR01010580.1_848156 (LRRC9), diagnostic of shore/beach spawners (Veale & Russello, 2017a). Interestingly, Hirst (1991) indicated that Gold Creek, a tributary of Alouette Reservoir (Figure 1), was the original spawning location for returning sockeye salmon, which was still accessible after the dam was constructed. However, dams often alter not only accessibility to the spawning grounds, but water temperature, food web dynamics, and quality of available habitat (Angilletta et al., 2008; Sheer & Steel, 2006). Human‐mediated changes to the Alouette watershed may have acted as a selection pressure or promoted plasticity in *O. nerka* spawning location, potentially driving a life‐history shift to deep‐spawning along the shoreline. In addition, genotypes at another outlier of interest, SZNR01024871.1_93859 (HSP90), revealed that all Alouette Reservoir individuals possessed the “G” allele, which has previously been reported for more than 95% of sockeye salmon distributed across multiple catchments (Columbia, Fraser) within British Columbia (Veale & Russello, 2017b).

We identified eight high confidence outlier loci between resident and migratory individuals in Alouette Reservoir, all of which mapped to linkage groups 9a and 9b that correspond to sockeye salmon sex chromosomes (Christensen et al., 2020). Unfortunately, sex information was not available for the migratory individuals genotyped in this study. Consequently, we were unable to disentangle associations of outlier loci and migratory behavior with those that could have been generated simply due to uneven sex ratios between the resident and migrant samples. Christensen et al. (2020) did note higher heterozygosity levels in male sockeye salmon compared to females at linkage groups 9a and 9b. In Alouette Reservoir, female residents and migrant *O. nerka* both demonstrated heterozygote deficiency at the outlier loci on linkage groups 9a and 9b, whereas male residents were largely heterozygous (mean *H_o_* = 0.93; Figure 3). Taken together, these patterns might serve as indirect evidence that the majority of migrant Alouette *O. nerka* in our study were female, and that the primary signal associated with outliers detected between Alouette Reservoir migrant and resident individuals was due to sex rather than life history. It is important to note, however, that reduced representation sequencing methods, such as RADseq, only capture a fraction of the genome: given that the sockeye salmon genome is estimated to be 2.6 Gbp (Christensen et al., 2020), the probability that a gene or genes underlying a certain phenotypic trait will be found among the several thousand examined markers is low. Consequently, a more comprehensive investigation of the genetic basis of life‐history variation in this system and others would be best served by future analyses of whole‐genome sequence data.

Overall, the lack of genetic distinctiveness between migrant and resident Alouette *O. nerka* at genome‐wide neutral loci, together with genotyping information at outlier loci, suggest that Alouette *O. nerka* represent a recently landlocked sockeye salmon population, as previously proposed (Godbout et al., 2011; Samarasin et al., 2017). This finding is significant, as it identifies Alouette Reservoir as the only known location where anadromous sockeye salmon may exhibit deep‐spawning behavior. Additionally, many of the characteristics exhibited by Alouette *O. nerka* are shared with residualized (i.e., resident progeny of anadromous parents) and partially migrating salmonids, particularly as they relate to sex ratios. First, Ricker (1938) documented the sex ratio of residuals as heavily skewed toward males relative to those observed in co‐occurring kokanee. A similar pattern was observed in Alouette Reservoir, where mature residents collected in 2018 were predominantly male (150M:29F). However, the unequal sex ratio could also be due to the timing of sampling, as male *O. nerka* in Alouette Reservoir tend to inhabit littoral regions prior to females (Hébert, 2019). Although not as common in *O. nerka*, skewed sex ratios are frequently reported in facultatively anadromous salmonid species, such as *Oncorhynchus mykiss*, where anadromy has been demonstrated to be maternally linked (Berejikian et al., 2014). Moreover, a chromosomal inversion on *Omy05* exhibits reversed sex‐dependent dominance in *O. mykiss*; in females, the ancestral karyotype that favors migration appears to be dominant, whereas in males, the pattern is reversed (Pearse et al., 2019). Similarly, in brown trout (*Salmo trutta*), resident populations tend to be predominantly male and anadromous populations predominantly female; however, those that do not have access to migration due to natural impediments may exhibit a more equal sex ratio (reviewed in Ferguson et al., 2017). The higher propensity of females to migrate can be attributed to the observation that in salmonids, female fecundity and reproductive success are directly proportional to size, and ocean‐rearing provides more resources for biomass accumulation. Consequently, it is typically in the best interest of females to maximize feeding potential by migrating to the ocean (Jonsson & Jonsson, 1993). Anadromous females are also typically larger and have higher fecundity than resident females (Kendall et al., 2015). In contrast, alternative strategies employed by males (e.g., sneaking) may decrease the importance of reaching a certain size to maximize reproductive success (Foote et al., 1997).

Other characteristics shared by Alouette *O. nerka* and residualized sockeye salmon are associated with spawning, including morphology and behavior. For example, Ricker (1938) described Cultus Lake residuals as exhibiting a dark olive/black coloration during the spawning period, similar to that observed in both Alouette Reservoir resident and returning migratory adults. Cultus Lake residuals were also found among redds of returning sockeye salmon during spawning season (Ricker, 1938). Likewise, in Alouette Reservoir, telemetry of returning adult upstream migrants and targeted netting of residents suggests both resident and migratory individuals spawn at the same depth (Hébert, 2019).

### 4.3 Management implications

Our study provides important information for guiding ongoing fisheries management operations for Alouette Reservoir. Specifically, our genome‐wide analyses revealed that Alouette Reservoir *O. nerka* represents a single stock that is likely best characterized as landlocked sockeye salmon, with individuals that retain the ability to migrate. As a consequence, efforts to provide passage to reinforce sockeye salmon in this system appear sound. Additionally, outlier analysis uncovered potential sex bias with respect to migration in this system, which, if validated, should also be considered in the context of sockeye salmon restoration efforts. In particular, strategies aiming to increase the number of migrating males (e.g., by controlling food availability) might be necessary in order to ensure a more even sex ratio among the returning adult migrants. Importantly, our results suggest that Alouette Reservoir may host the only known population of anadromous sockeye salmon that spawn at depth, punctuating the need for a reassessment of its conservations status, which is currently considered by the Committee on the Status of Endangered Wildlife in Canada to be extirpated (COSEWIC, 2017). Moreover, this work highlights the value of the Alouette system for future investigation of ecological and evolutionary questions associated with the impacts of water control structures on anadromous species.

## CONFLICT OF INTEREST

None declared.

## AUTHOR CONTRIBUTIONS

**Farida Samad‐zada:** Conceptualization (supporting); Data curation (lead); Formal analysis (lead); Investigation (lead); Writing‐original draft (lead); Writing‐review & editing (equal). **Brett T. van Poorten:** Conceptualization (supporting); Funding acquisition (supporting); Resources (supporting); Supervision (supporting); Writing‐review & editing (supporting). **Shannon Harris:** Conceptualization (supporting); Resources (supporting); Writing‐review & editing (supporting). **Lyse Godbout:** Resources (supporting); Writing‐review & editing (supporting). **Michael A. Russello:** Conceptualization (lead); Funding acquisition (lead); Investigation (supporting); Project administration (lead); Resources (lead); Supervision (lead); Writing‐original draft (supporting); Writing‐review & editing (equal).

## Data Availability

All Illumina raw reads are available from the NCBI sequence read archive (BioProject ID: PRJNA752800). RAD tag sequences, SNP genotypic data, and population phenotypic data are deposited in DRYAD (https://doi.org/10.5061/dryad.dz08kprz2).

## APPENDIX A

**TABLE A1:** Populations, morphs and spawner types of *Oncorhynchus nerka* populations examined in this study

Population	Morphs	Spawner type	NS	NR	NF	Source
Alouette Reservoir	Resident	Deep‐spawner	68	8	61	New data
	Adult migrant	Deep‐spawner^a^	85	7	78	
	Juvenile migrant	Deep‐spawner^a^	26	0	24	New data
Scotch Creek	Sockeye	Stream‐spawner	25	2	25	New data
Portage Creek	Sockeye	Stream‐spawner	NA	NA	23	Veale and Russello (2017b)
Anderson Lake	Kokanee	Deep‐spawner	NA	NA	22	Veale and Russello (2017b)
Seton Lake	Kokanee	Deep‐spawner	NA	NA	23	Veale and Russello (2017b)
East Barrière Lake	Kokanee	Deep‐spawner	31	2	31	New data
Nicola Lake	Kokanee	Stream‐spawner	25	0	25	New data
Total			260		312	

Abbreviations: NF; NR, number of replicates; NS, number of unique samples sequenced; number of unique samples retained after filtering.

^a^ See Section 1 for spawning information of migrant Alouette *O. nerka*.

**TABLE A2:** Sensitivity analysis to identify the optimal set of parameters for the *populations* run (Stacks v2.41; Catchen et al., 2011)

*r*	min_maf	Number of samples (Depth > 6×)	SNPs	Mean missing %	Mean depth
0.7	0.01	330	14,948	4.1	24.58
	0.03	330	11,313	4.5	24.75
	0.05	330	9,700	4.75	24.75
0.75	0.01	330	14,257	3.74	24.76
	0.03	330	10,752	4.11	24.91
	0.05	330	9,220	4.37	24.92
0.8	0.01	330	13,213	3.26	25.06
	0.03	330	9,899	3.61	25.24
	0.05	330	8,458	3.84	25.27
0.85*	0.01	330	11,805	2.74	25.48
	0.03	330	8,723	3.05	25.72
	0.05*	330	7,390	3.25	25.79
0.9	0.01	330	9,153	1.96	26.23
	0.03	330	6,516	2.18	26.59
	0.05	330	5,355	2.35	26.8
0.95	0.01	330	5,233	1.08	27.52
	0.03	330	3,310	1.22	28.03
	0.05	330	2,471	1.3	28.47

Note

Optimal set of parameters indicated with an asterisk.

**TABLE A3:** IDs and annotations of the eight high‐confidence outlier loci detected between Alouette Reservoir resident and migrant *Oncorhynchus nerka*

SNP	Annotation
SZNR01019698.1_742835	NA
SZNR01029840.1_674118	PREDICTED: *Oncorhynchus kisutch* TIR domain containing adaptor protein (tirap), mRNA
SZNR01029840.1_812756	NA
SZNR01029840.1_1007455	PREDICTED: *Oncorhynchus nerka* phosphatidylinositol 3‐kinase regulatory subunit gamma‐like (LOC115114221), transcript variant X3, mRNA 1
SZNR01029840.1_1168439	PREDICTED: *Oncorhynchus kisutch* proteoglycan 4 (LOC109908567), mRNA
SZNR01032361.1_1519020	PREDICTED: *Oncorhynchus kisutch* thioredoxin domain containing 16 (txndc16), transcript variant X1, mRNA
SZNR01034902.1_2245482	NA
SZNR01032361.1_2116325	NA

**TABLE A4:** Evanno Table generated using STRUCTURE Harvester, showing Ln'(*K*) and Delta *K* values, based on 10 iterations of STRUCTURE output, with the number of clusters (*K*) varying from 1 to 10

# *K*	Reps	Mean LnP(K)	Stdev LnP(K)	Ln'(K)	|Ln''(K)|	Delta K
1	10	−1,963,925	10.6608	NA	NA	NA
2	10	−1,782,303	9.3031	181,621.3	139,275.4	14,970.9
3	10	−1,739,957	334.5973	42,345.87	5,070.43	15.15383
4	10	−1,702,682	12,741.36	37,275.44	8,329.22	0.653715
5	10	−1,673,736	59.529	28,946.22	28,137.88	472.6752
6	10	−1,672,927	948.4455	808.34	32,638.89	34.41304
7	10	−1,704,758	93,309.67	−31,830.6	13,395.52	0.14356
8	10	−1,749,984	225,893.5	−45,226.1	33,303.29	0.147429
9	10	−1,761,907	270,804.9	−11,922.8	217,664	0.803767
10	10	−1,991,494	527,410.6	−229,587	NA	NA
